# Intervertebral disc cells as competent phagocytes *in vitro*: implications for cell death in disc degeneration

**DOI:** 10.1186/ar2466

**Published:** 2008-08-01

**Authors:** Philip Jones, Lucy Gardner, Janis Menage, Gwyn T Williams, Sally Roberts

**Affiliations:** 1 Centre for Spinal Studies, Robert Jones & Agnes Hunt Orthopaedic & District Hospital NHS Trust, Oswestry, Shropshire SY10 7AG, UK; 2 Institute of Science and Technology in Medicine, Keele University, Keele, Staffordshire, ST5 5BG, UK

## Abstract

**Introduction:**

Apoptosis has been reported to occur in the intervertebral disc. Elsewhere in the body, apoptotic cells are cleared from the system via phagocytosis by committed phagocytes such as macrophages, reducing the chance of subsequent inflammation. These cells, however, are not normally present in the disc. We investigated whether disc cells themselves can be induced to become phagocytic and so have the ability to ingest and remove apoptotic disc cells, minimising the damage to their environment.

**Method:**

Bovine nucleus pulposus cells from caudal intervertebral discs were grown in culture and exposed to both latex particles (which are ingested by committed phagocytes) and apoptotic cells. Their response was monitored via microscopy, including both fluorescent and video microscopy, and compared with that seen by cell lines of monocytes/macrophages (THP-1 and J774 cells), considered to be committed phagocytes, in addition to a nonmacrophage cell line (L929 fibroblasts). Immunostaining for the monocyte/macrophage marker, CD68, was also carried out.

**Results:**

Disc cells were able to ingest latex beads at least as efficiently, if not more so, than phagocytic THP-1 and J774 cells. Disc cells ingested a greater number of beads per cell than the committed phagocytes in a similar time scale. In addition, disc cells were able to ingest apoptotic cells when cocultured in monolayer with a UV-treated population of HeLa cells. Apoptotic disc cells, in turn, were able to stimulate phagocytosis by the committed macrophages. CD68 immunostaining was strong for THP-1 cells but negligible for disc cells, even those that had ingested beads.

**Conclusion:**

In this study, we have shown that intervertebral disc cells are capable of behaving as competent phagocytes (that is, ingesting latex beads) and apoptotic cells. In terms of number of particles, they ingest more than the monocyte/macrophage cells, possibly due to their greater size. The fact that disc cells clearly can undergo phagocytosis has implications for the intervertebral disc *in vivo*. Here, where cell death is reported to be common yet there is normally no easy access to a macrophage population, the endogenous disc cells may be encouraged to undergo phagocytosis (for example, of neighbouring cells within cell clusters).

## Introduction

Cells are the vital machinery for synthesising and maintaining the functioning matrix in all tissues and the intervertebral disc within the spine is no different. Cell death within the disc cell population has been reported to be a common phenomenon and recently there have been several studies showing that apoptosis, or controlled cell death, occurs here [1-7]. Apoptosis is a genetically controlled mechanism that is considered to be important for tissue homeostasis. The cell dies in a well-defined process involving condensation of the chromatin and packaging of cell components within lipid membranes to form apoptotic bodies, thus minimising any subsequent damage to the surrounding matrix [8,9]. This is in contrast to necrosis, which is relatively uncontrolled with the cell membrane disrupting and releasing cellular contents. Necrosis is believed to be more damaging to the tissue with the release of degradative enzymes and the ability to illicit an inflammatory response [10].

Apoptosis is often described as a 'silent death' [11] with cells being destroyed from within [12] and the remains of the cell subsequently 'eaten' by phagocytic cells, effectively eliminating all physical evidence of death. In most tissues, this clearance of apoptotic cells will be undertaken by the committed phagocytes of the macrophage lineage, available via the local blood supply. However, the normal adult intervertebral disc has little or no direct vasculature supplying it, particularly the central nucleus pulposus (NP) [13], where cell death is reported to be most common [14]. This raises the question of how apoptotic cells within the intervertebral disc might be cleared. Other cell types have been reported to be induced to phagocytose when exposed to stimuli if macrophages are not available (for example, epithelial, endothelial, and tumour cells) [15]. The mechanism is not fully understood, but dying cells appear to elicit 'eat me' signals (for example, exposure of a phosphatidylserine molecule on the outer surface of the cell membrane [16] which can stimulate other cells to become phagocytic, albeit as facultative phagocytes).

We hypothesised that intervertebral disc cells could behave in this manner and that, if exposed to appropriate stimuli such as apoptotic cells, they could be induced to become phagocytic. This *in vitro *study, comparing the response of bovine NP cells with that of committed phagocytes to exposure both to latex beads (a commonly used stimulus for phagocytosis) and to apoptotic cells, has demonstrated this to be the case.

## Materials and methods

### Nucleus pulposus cell extraction and cell lines

NP was dissected from the centre of the three uppermost bovine caudal discs obtained from young adult cattle (n = 15, ages 18 to 32 months) within 1 hour of death with permission from a local abattoir. The tissue of the three discs was pooled and the NP cells were isolated by incubating the diced tissue overnight at 37°C in 0.8 mg/mL crude type XI collagenase (Sigma-Aldrich, Gillingham, Dorset, UK) containing 1.67 units per millilitre DNase (Sigma-Aldrich). The cells obtained after digestion were washed using Dulbecco's modified Eagle's medium (DMEM)/F-12 (Invitrogen Corporation, Paisley, UK) supplemented with 10% foetal bovine serum (FBS) (PAA Laboratories, Yeovil, Somerset, UK) and were centrifuged at 107 *g *for 10 minutes. The cells were then filtered through a 70-μm nylon cell strainer (BD Biosciences, Cowley, UK). The extracted cells were grown in monolayer culture in DMEM/F-12 in a fully humidified atmosphere with 5% CO_2 _and 21% O_2 _at 37°C. The cells were expanded and passaged twice before use.

Two cell lines, THP-1 and J774 cells, were used as committed phagocytes whilst the fibroblast cell line, L929, was used for comparison as a nonmacrophage-like cell and HeLa was used to provide a source of apoptotic cells. The human monocytic leukaemic cell line, THP-1 [17] (European Collection of Cell Cultures [ECACC], Salisbury, UK), was maintained in RPMI 1640 medium (Invitrogen Corporation) supplemented with 10% FBS. The J774 cell line (derived from murine macrophages, kindly donated by Robin May, University of Birmingham, UK) was maintained in DMEM/F-12 supplemented with 10% FBS. HeLa cells (donated by Mann Nguyen, Robert Jones & Agnes Hunt Orthopaedic & District Hospital NHS Trust, Oswestry, Shropshire, UK), an immortalised epithelial cell line, were also maintained in RPMI 1640 supplemented with 10% FBS. L929, a mouse fibroblast cell line, was obtained from ECACC (number 85011425) and cells were grown in DMEM/F-12 and 10% FBS. This study does not involve human subjects, human tissue, or experimentation on animals.

### Phagocytosis assays using latex beads

**Figure 1:**
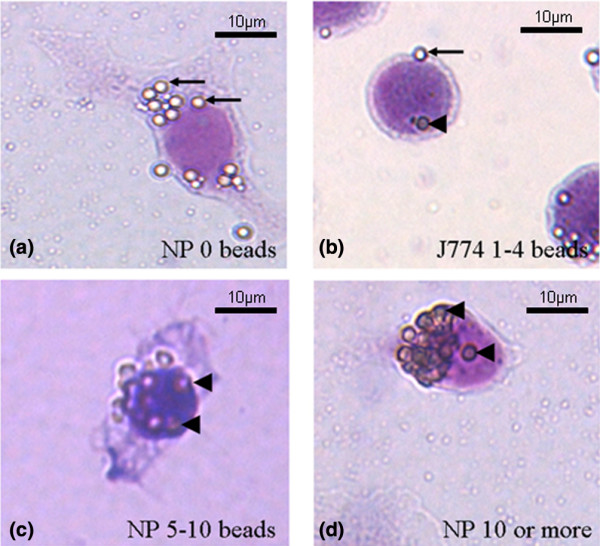
Phagocytosis of latex beads by intervertebral disc and other cell types. Bovine nucleus pulposus (NP) cells or J774 cells that ingested **(a) **0 latex beads, **(b) **1 to 4 beads, **(c) **5 to 10 beads, and **(d) **more than 10 beads are shown (Jenner-Giemsa stain). Beads outside the cell membrane are clearly more birefringent and brighter (arrows) than those ingested, which have a dull appearance (arrowhead).

To activate the THP-1 to a macrophage phenotype, the cells were treated with 160 nM phorbol 12-myristrate 13-acetate (PMA) (Sigma-Aldrich) for 72 hours [18]. The cells were washed with RPMI and then incubated with RPMI containing 0.02% 2-μm-diameter fluorescein isothiocyanate (FITC)-latex beads (Sigma-Aldrich). Alternatively, to activate the J774 cells to a macrophage phenotype, they were treated with 243 nM PMA for 2 hours, washed with DMEM/F-12, and then incubated with DMEM/F-12 containing 0.02% 2-μm-diameter FITC-latex beads [19]. NP cells were grown in monolayer culture for 24 hours before adding latex beads as above. Cells were grown at a concentration of 500,000 cells per well in six-well plates. At time points between 0 and 48 hours (approximately 1, 2, 4, 6, 8, 24, 32, and 48 hours), cultures were fixed in methanol for 5 minutes, before staining with Jenner-Giemsa stain [20]. Two hundred cells for each culture were observed and the number counted which had ingested any latex beads in addition to the number of cells (a) that had not ingested any beads and that had ingested (b) 1 to 4 beads, (c) 5 to 10 beads, and (d) more than 10 beads (Figure 1). All cultures for each set of measurements were done in quadruplicate.

### Phagocytosis of apoptotic HeLa and nucleus pulposus cells by THP-1 and J774 cells

**Figure 2:**
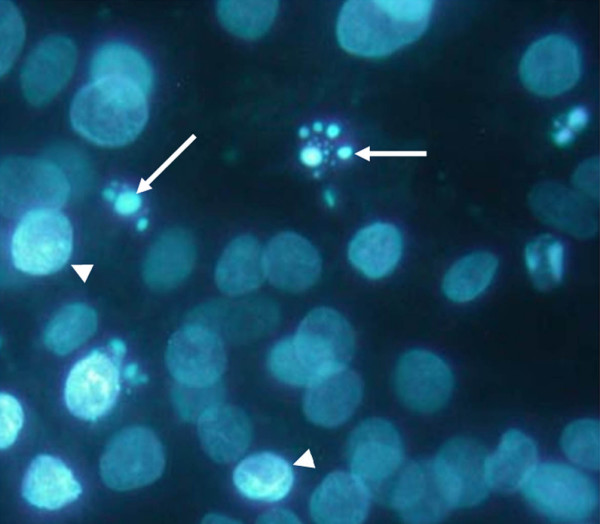
Morphology of apoptotic disc cells. Hoechst 33342-stained nucleus pulposus cells demonstrate typical apoptotic morphology after 3 minutes of UV treatment: condensation of the nuclear material (arrowheads) followed by formation of apoptotic bodies (arrows). Original magnification: ×630.

Both THP-1 and J774 cells were fluorescently tagged using a green fluorescent cell linker mini kit (catalogue number MINI-67; Sigma-Aldrich) [21] and then pretreated with PMA as described above. Cells to be induced to become apoptotic, whether NP or HeLa cells, were fluorescently tagged using a red fluorescent cell linker mini kit (catalogue number MINI-26; Sigma-Aldrich). NP or HeLa cells (1 × 10^6^) in 2 mL/well were tagged. Apoptosis was induced in the NP and HeLa cells by exposing them to UV light (UVG54 grid lamp; UVP Ltd, Cambridge, Cambridgeshire, UK) at a distance of 15 cm for 3 minutes [22], providing a UV dose of approximately 560 W/cm^2^. This has been shown to cause approximately 50% of the cells to become apoptotic (as can be seen with Hoechst 33342 dye, Figure 2). These cells were then added to the activated THP-1 or J774 cells at a ratio of 2 UV-treated cells to 1 normal cell. Time-lapse video microscopy using a digital video camera (TK-1280E; JVC, Yokohama, Japan) was used to capture images every 5 minutes over the span of 72 hours. The digital images were then converted into video files using Media Studio Video Editor (version 3.5; Ulead System Inc., Karst, Germany). Cells were also observed by means of a fluorescent microscope (Leica DMBL; Leica Microsystems GmbH, Wetzlar, Germany) after 24, 48, and 72 hours. Images were taken using IPLab Scientific Imaging Software (Scanalytics Inc., part of BD Biosciences).

### Phagocytosis of apoptotic cells by nucleus pulposus cells

NP cells were fluorescently tagged red with a cell linker kit (Sigma-Aldrich) and allowed to adhere overnight. NP cells (1 × 10^5^) in 2 mL/well were used. HeLa cells (5 × 10^5 ^per well) were fluorescently tagged green and exposed to UV light for 3 minutes as above. They were then added to the NP cells and left for up to 72 hours. Time-lapse video microscopy and fluorescent images were taken at 24, 48, and 72 hours and used to observe the two populations within the cocultures, red-labelled NP cells in monolayer with the green-labelled HeLa cells (apoptotic).

### CD68 immunostaining

Immunostaining for CD68 was carried out on bovine NP cells cultured both with and without latex beads for 0, 4, 6, and 24 hours, in addition to THP-1 cells, pretreated with PMA, and grown on coverslips for 24 hours. Slides were fixed in acetone and incubated with SSC (150 mM sodium chloride and 15 mM sodium citrate at 55°C). Endogenous peroxidase was blocked with 0.3% hydrogen peroxide in methanol and further blocking was performed by incubating the sections with normal serum. Sections were then incubated with an antibody to CD68 (1:23 in phosphate-buffered saline; Dako, Glostrup, Denmark, clone EBM11). Labelling was visualised with peroxidase and diaminobenzadine as the substrate and enhanced with avidin-biotin complex (Vector Laboratories, Burlingame, CA, USA). Mouse IgG was used as a negative control. Immunopositivity was assessed by recording at least 200 cells for each cell culture at each time point.

## Results

### Response of committed phagocytes and fibroblasts to exposure to latex beads

**Figure 3:**
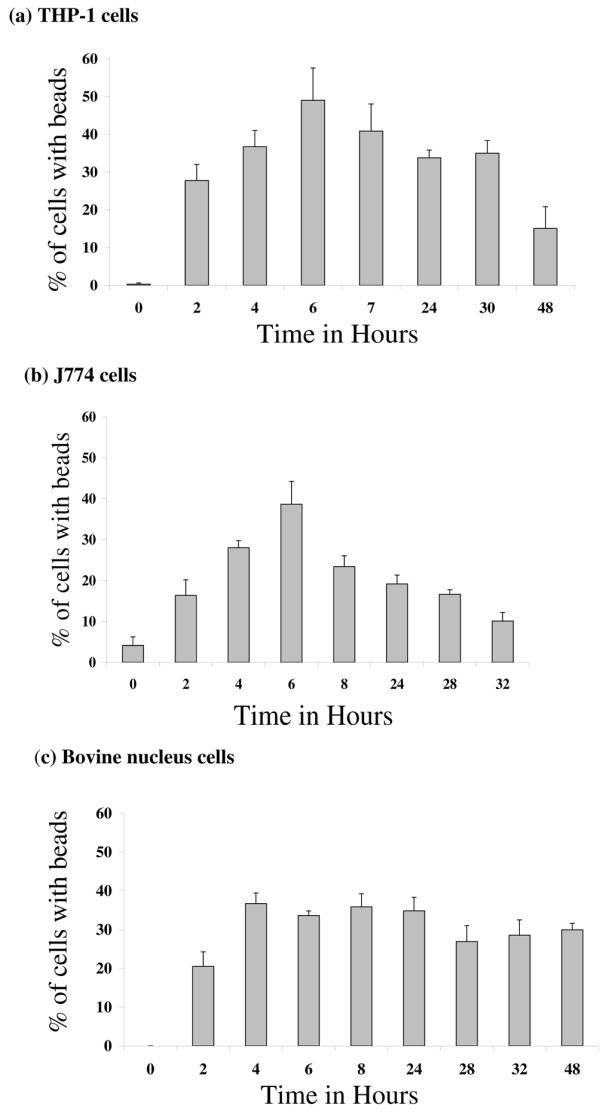
Frequency of beads ingested by cells. Bar charts present the percentage of cells that had ingested beads between 0 and 48 hours after the addition of latex beads for all three cell types investigated: **(a) **THP-1, **(b) **J774, and **(c) **nucleus pulposus cells. Bar indicates standard error (n = 4 cultures for each time point).

**Figure 4:**
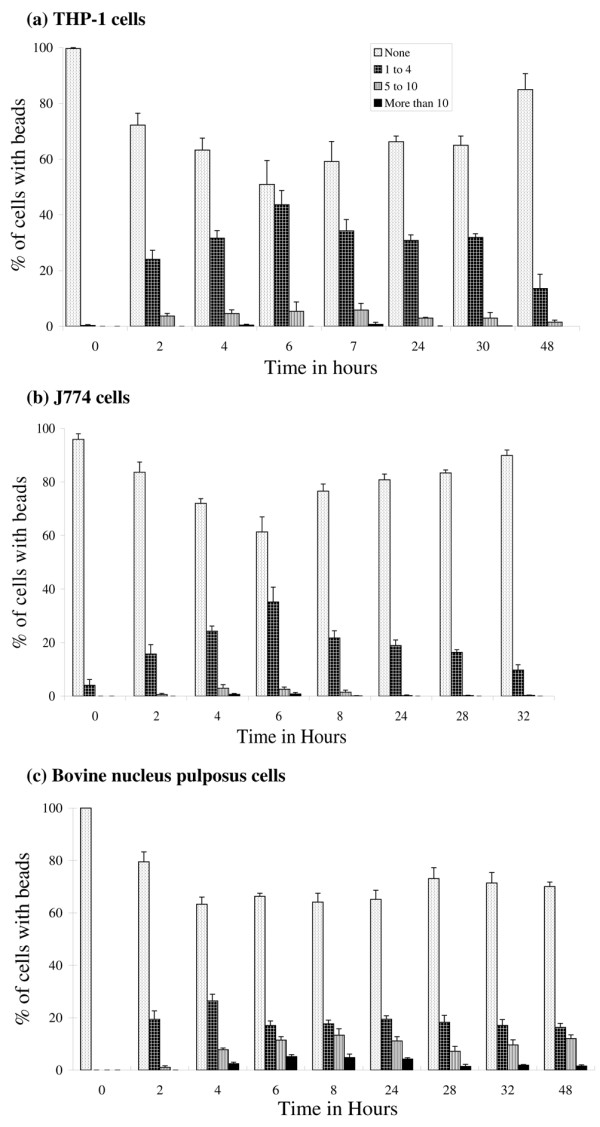
Numbers of beads ingested by cells. Frequency bar chart shows the percentage of cells that had ingested different numbers of latex beads with time: **(a) **THP-1 cells, **(b) **J774 cells, and **(c) **nucleus pulposus cells. Bar indicates standard error (n = 4 cultures for each time point).

PMA-activated THP-1 and J774 cells responded to being cultured with latex beads, by ingesting significant numbers of them with time, as previously described [18]. Two hours after exposure, 27.8% and 16.4% of THP-1 and J774 cells, respectively, had ingested at least some beads, with maximum ingestion at around 6 hours for both cell types (Figure 3a and 3b). Forty-nine percent of THP-1 cells and 38.6% of J774 cells had ingested beads at this time point, with 5.4% and 1.7%, respectively, having ingested more than four beads per cell (Figures 4a and 4b). At time points beyond 6 hours, the number of both cell types with obvious beads ingested reduced. In contrast to the committed phagocytes and NP cells (see below), only 2.5% of L929 fibroblast cells had ingested any beads at 6 hours, and only 0.1% of them with more than four beads per cell.

### Response of nucleus pulposus cells to exposure to latex beads

NP cells also responded to being cultured with latex beads by ingesting them. After 2 hours, 20.5% of cells had ingested some beads, but this increased to a maximum of 36.7% at 4 hours, after which the number decreased slightly with time, but not to levels as low as seen for THP-1 or J774 cells (Figure 3c). At 6 hours, 16.7% of NP cells had ingested more than four beads per cell (Figure 4c).

### Phagocytosis of apoptotic cells by committed phagocytes and nucleus pulposus cells

**Figure 5:**
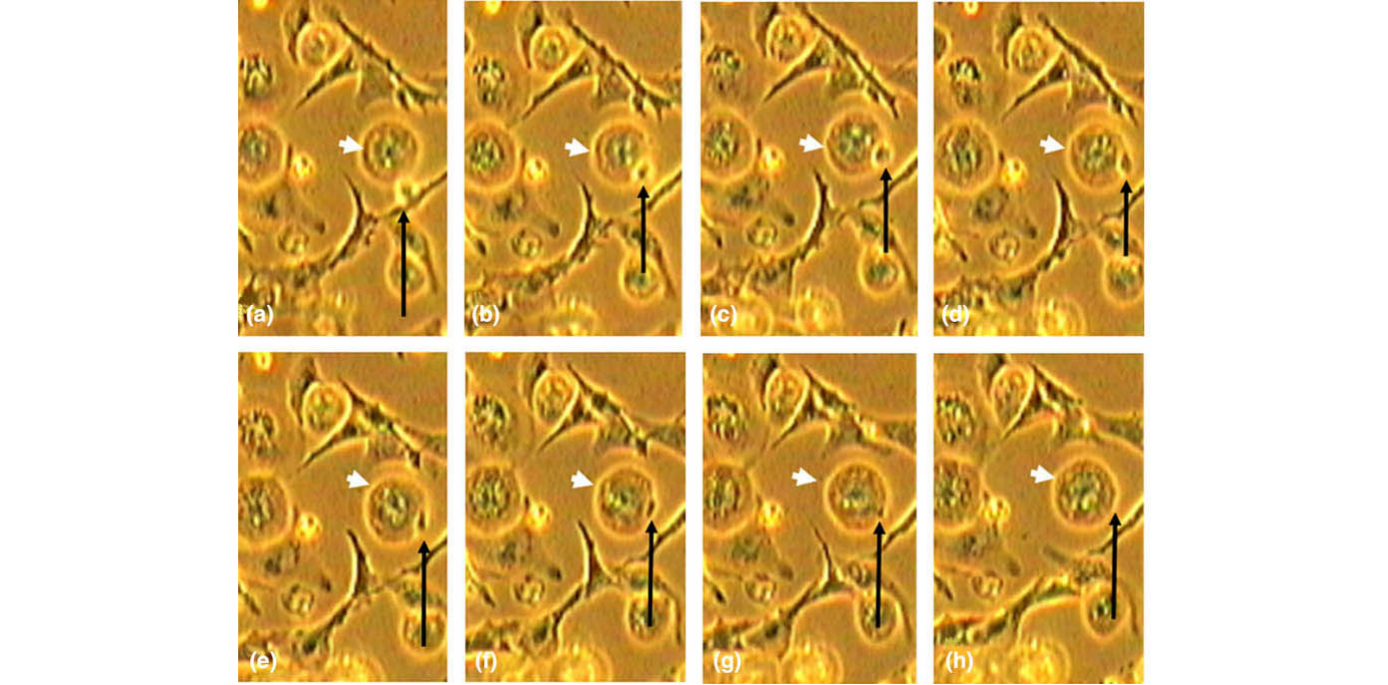
Apoptotic disc cells stimulating phagocytosis by other cells. A sequence of phase-contrast images obtained from video microscopy shows a THP-1 cell (white arrow) ingesting an apoptotic, phase-bright nucleus pulposus cell (black arrow). These frames demonstrate that apoptotic disc cells can trigger a phagocytic response. Images were taken over the course of 40 minutes. Original magnification × 100.

**Figure 6:**
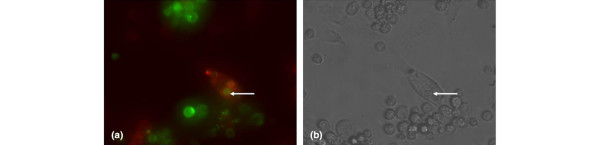
Intervertebral disc cells stimulated to undergo phagocytosis. Fluorescently tagged (red) bovine nucleus pulposus cells ingest fluorescently tagged (green) apoptotic HeLa cells: **(a) **fluorescent image and **(b) **phase-contrast image of the same field. This sequence demonstrates that disc cells are capable of phagocytosing apoptotic cells. Arrow shows ingested apoptotic body. Original magnification × 400.

Coculturing of both the committed phagocytes, J774 and THP-1 cells, with HeLa or NP cells that had been UV-treated to render them apoptotic was monitored by video microscopy. It was possible, over a period of several hours, to follow certain J774 and THP-1 cells as they moved around the tissue culture flasks, made contact with an apoptotic cell or apoptotic body, and subsequently ingested it. Such a sequence demonstrating this activity with apoptotic disc cells has been separated into individual still shots and is shown in Figure 5, demonstrating that apoptotic disc cells can stimulate their phagocytosis by the committed macrophages. Similarly, NP cells cocultured with UV-treated apoptotic cells could be seen to phagocytose and ingest the apoptopic cells (Figure 6). This demonstrated that disc cells can be 'switched on' to phagocytose apoptopic cells (Figure 6).

### CD68 immunostaining

**Figure 7:**
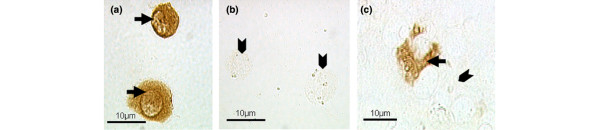
CD68 immunostaining of THP-1 and bovine nucleus pulposus cells. **(a) **Virtually all THP-1 cells were positively immunostained with the CD68 antibody (arrows) and **(b) **negatively immunostained with the normal mouse IgG antibody (arrowheads). **(c) **There were very few positively stained bovine nucleus pulposus cells for CD68 (arrow). Most were negative (arrowhead).

Immunopositivity for CD68 was seen in none of the NP cells without exposure to latex beads and in very few cells exposed to beads (<2% of cells). In contrast, greater than 95% of THP-1 cells were immunopositive (Figure 7).

## Discussion

Removal of apoptotic cells by phagocytes is considered to be the final common event in the life of most apoptotic cells [23]. Efficient clearance before lysis occurs is critical to tissue health and integrity. Phagocytic clearance of apoptotic cells by macrophages or facultative phagocytic cells constitutes an integral part of the overall suicide process [24] and is thought to provide a safe disposal route [25]. In addition, it can actively downregulate inflammatory responses.

Whilst apoptosis of cells within the intervertebral disc has been investigated by several groups, little attention has been paid to the subsequent physiological process and clearance of the apoptotic cells. The frequency of apoptosis is unclear, with 50% to 70% of cells being reported as apoptotic by some workers [2] (or at least as being TUNEL [terminal deoxynucleotidyl transferase-mediated dUTP-biotin nick end-labeling]-positive), but thought to be considerably less by others [26]. Whatever the incidence, the presence of apoptotic cells would not cause concern if the disc were a vascularised tissue where macrophages could eliminate such cells from the environment. However, the adult healthy disc is considered to be the largest avascular tissue in the body [27], so the accessibility of macrophages is likely to be more limited than in most tissues. Other cell types, including fibroblasts, glomerular mesangial cells, endothelial cells, and even chondrocytes, have been reported to be capable of phagocytic ingestion of apoptotic cells of the same lineage in the absence of committed phagocytes [15,16,28].

Molecules that may trigger engulfment by committed phagocytes have been identified to some extent (including phosphatidylserine, recognised by receptors such as CD36, CD68, or CD14), but much less is known about the molecular cascade that may occur with the 'amateur' or facultative phagocytes [15,23]. Lectins have been suggested to be more important in facultative phagocytes than the committed population [16]. It is suggested that phagocytosis of apoptotic cells, however it occurs, may be beneficial to the tissue in more ways than one. In addition to removing potential matrix-degrading enzymes, phagocytosis can trigger the release of anti-inflammatory cytokines such as transforming growth factor-beta whilst inhibiting the production of proinflammatory cytokines, including tumour necrosis factor-alpha [11].

Whilst macrophages or mononuclear cells have been reported to occur in herniated extruded intervertebral discs [29], they are not observed in healthy discs. Markers typical of macrophages have been reported in cells within the intervertebral disc. Virri and colleagues [30] have described 55% of herniated human discs to contain CD68-immunopositive cells, but only in areas close to blood vessels. Nerlich and colleagues [31] found no CD68-positive cells in discs of foetus, infants, or adolescents. In contrast, some CD68-positive cells were seen in the NP of all discs showing disc degeneration, but the morphology of these cells was no different from that of the cells normally found here. Thus, the authors suggest that the CD68-positive cells are not invaded monocytes or macrophages but transformed resident cells that are involved in phagocytosis. Our study would support this and provides evidence that NP disc cells can indeed behave as phagocytes and undertake phagocytosis, at least *in vitro*. Indeed, they are able to ingest more latex beads than the committed phagocytes, although this may be, in part, a reflection of their larger size (disc cells had a mean area of 544 ± 135 μm^2 ^compared with 160 ± 93 μm^2 ^for THP-1 cells and 175 ± 49 μm^2 ^for J774 cells in monolayer). In addition, the disc cells appeared to be able to retain the beads that they had ingested better than the committed phagocytes, in which the number of beads internalised decreased with time, perhaps due to subsequent extrusion of some beads, as has been seen by macrophages previously [32]. Disc cells were certainly much more effective at ingesting the beads than the fibroblast cell line, L929. Cells in other cartilaginous tissue are also reported to be capable of becoming phagocytic; for example, articular cartilage chondrocytes have been shown to behave in a similar manner [28] and occasionally the cells in epiphyseal cartilage [33]. The lack of CD68 expression by the disc cells in this study may be a feature of *in vitro *culture as CD68 has been shown to be expressed by osteoblasts with increasing time in culture, independently of how many particles had been phagocytosed [34]. However, in the study on osteoblasts, CD68 was found after 72 hours in culture (with no earlier observations); in the current study, NP cells were cultured for a maximum of only 48 hours.

The capability of disc cells to phagocytose and the capability of apoptopic disc cells to stimulate phagocytosis could be beneficial to the intervertebral disc *in vivo *in many ways. Death of the cells within the intervertebral disc is reported to be extensive [35] and increasingly common after the age of 11 in humans [36]. Efficient clearance of the dying cells, whether they have died by apoptosis or autophagy, is advantageous. If they are not cleared, it may lead to secondary necrosis with its subsequent harmful impact on the cells' environment [24] and possible stimulation of an inflammatory response. It also provides a means of clearing senescent cells, of which there are plenty in the intervertebral disc, particularly in herniated discs [37] or degenerate discs [38,39]. Although disc cells often occur in isolation with much matrix around them, in degenerate discs, clusters of cells, with individual cells directly contacting their neighbours, are a common feature. Whilst migration of disc cells through the matrix may be an unlikely phenomenon, phagocytosis of neighbouring or adjacent cells in clusters would appear to be perfectly feasible.

## Conclusion

In summary, this study shows that bovine NP cells are able of phagocytosing latex beads and apoptotic bodies. The pattern of phagocytosis of beads is comparable to the pattern shown by the committed phagocytes, THP1 and J774 cell lines. This, together with earlier reports that disc cells can express CD68, a phagocytic marker [31], suggests that the cells of the intervertebral disc may be capable of the removal of apoptotic bodies when disc cells die *in vivo *via apoptosis or indeed any variation such as autophagy [24]. This could have very important physiological implications since removal of apoptotic cells is likely to be the most important event *in vivo *if tissue structure and function are to be maintained in the face of major cell loss [23].

## Abbreviations

DMEM = Dulbecco's modified Eagle's medium; ECACC = European Collection of Cell Cultures; FBS = foetal bovine serum; FITC = fluorescein isothiocyanate; NP = nucleus pulposus; PMA = phorbol 12-myristrate 13-acetate.

## Competing interests

The authors declare that they have no competing interests.

## Authors' contributions

PJ and LG carried out the practical laboratory work. JM assisted with data analysis and writing the manuscript. GTW provided expert advice. SR raised funds, conceived the research, and wrote the manuscript. All authors read and approved the final manuscript.
